# Predictors of mortality of patients with acute respiratory failure secondary to chronic obstructive pulmonary disease admitted to an intensive care unit: A one year study

**DOI:** 10.1186/1471-2466-4-12

**Published:** 2004-11-27

**Authors:** GC Khilnani, Amit Banga, SK Sharma

**Affiliations:** 1 Department of Medicine, All India Institute of Medical Sciences, New Delhi-110029, India

## Abstract

**Background:**

Patients with acute exacerbation of chronic obstructive pulmonary disease (COPD) commonly require hospitalization and admission to intensive care unit (ICU). It is useful to identify patients at the time of admission who are likely to have poor outcome. This study was carried out to define the predictors of mortality in patients with acute exacerbation of COPD and to device a scoring system using the baseline physiological variables for prognosticating these patients.

**Methods:**

Eighty-two patients with acute respiratory failure secondary to COPD admitted to medical ICU over a one-year period were included. Clinical and demographic profile at the time of admission to ICU including APACHE II score and Glasgow coma scale were recorded at the time of admission to ICU. In addition, acid base disorders, renal functions, liver functions and serum albumin, were recorded at the time of presentation. Primary outcome measure was hospital mortality.

**Results:**

Invasive ventilation was required in 69 patients (84.1%). Fifty-two patients survived to hospital discharge (63.4%). APACHE II score at the time of admission to ICU {odds ratio (95 % CI): 1.32 (1.138–1.532); p < 0.001} and serum albumin (done within 24 hours of admission) {odds ratio (95 % CI): 0.114 (0.03-0.432); p = 0.001}. An equation, constructed using the adjusted odds ratio for the two parameters, had an area under the ROC curve of 91.3%. For the choice of cut-off, sensitivity, specificity, positive and negative predictive value for predicting outcome was 90%, 86.5%, 79.4% and 93.7%.

**Conclusion:**

APACHE II score at admission and SA levels with in 24 hrs after admission are independent predictors of mortality for patients with COPD admitted to ICU. The equation derived from these two parameters is useful for predicting outcome of these patients.

## Background

Chronic obstructive pulmonary disease (COPD) is characterized by irreversible airway obstruction that leads to chronic disability. Patients with COPD have a longstanding downhill course that is interspersed with episodes of exacerbations requiring hospitalization. COPD is known to be a common disease. There is lack of recent data regarding the burden of this disease from India, with only study on prevalence of COPD published in 1981 [1]. Data from United States indicate that incidence of disease is on the rise [2]. During the year 2000, approximately 24 million adults in United States had evidence of obstructive airway disease. COPD was responsible for 1.5 million emergency department visits, 726,000 hospitalizations, and 119,000 deaths [2]. It is obvious that this disease puts an enormous economic burden on the society. Andersson and coworkers estimated that almost 35-45% of the total per capita health-care costs for COPD are account for by exacerbations alone [3]. Severe exacerbations requiring hospitalizations are responsible for a large share of these costs and among these, treatment cost for those who require intensive care unit (ICU) admission is highest. In most of the third world countries, large number of ICU beds are occupied by patients with critical illnesses secondary to various infectious diseases, most of which are reversible.

It is important to identify patients at the time of admission who are likely to have poor outcome, so that such patients can be managed aggressively. Many prognostic scoring systems have been devised for the same purpose. These scoring systems help to segregate patients who are the sickest and are likely to die from those who are expected to have better outcome and survive. Most of these scoring systems have been devised for a broad range of critically ill patients. The present study was planned to determine the predictors of mortality in patients with exacerbation of COPD admitted to ICU over a one-year period. An attempt was made to develop a scoring system using the predictors of mortality that would help to identify patients at high risk of dying.

## Methods

Prospectively collected data of patients with acute respiratory failure secondary to COPD admitted to medical ICU of All India Institute of Medical sciences, New Delhi, India (a tertiary care center in north India) over a one-year period (January 2002 to December 2002) was reviewed. Diagnosis of COPD was based upon the characteristic findings on history and examination with typical radiographic abnormalities [4]. Patients admitted to the ICU with COPD but due to any other primary reason such as those with poisoning or acute coronary event were excluded. Similarly, patients in whom the primary cause of respiratory failure was bronchiectasis, bronchial asthma, pulmonary edema or pulmonary embolism were not included. Finally, 82 patients with a primary admission diagnosis of acute respiratory failure secondary to COPD were included. All patients were documented cases with prior pulmonary function test confirmation of irreversible airway obstruction and had been receiving a combination of various bronchodilators.

Management of the patients was the primary responsibility of the ICU team. A treatment strategy was individualized for each patient and was the sole prerogative of the treating physician. All patients received regular nebulized bronchodilators including salbutamol (as frequently as 5 mg every 15 minutes to every 8 hours), ipratropium bromide (as frequently as 0.5 mg every 15 minutes to 0.25 mg every 8 hours), and intravenous corticosteroids. Most patients also received antibiotics (n = 75, 91.5%). Oxygen therapy (2-3 lt/min) was administered to spontaneously breathing patients. The decision to institute ventilatory support was taken by the treating physician. Wherever feasible non-invasive ventilation (NIV) was used as the initial strategy. Endotracheal intubation was done for usual indications such as respiratory arrest, deteriorating level of consciousness, rising PaCO_2 _despite maximal pharmacological treatment and deteriorating acidemia. Initiation of weaning from mechanical ventilation was considered as soon as the patients were considered capable of breathing spontaneously. Method of weaning trials included t-piece trials, gradual reduction of synchronized intermittent mandatory ventilation (SIMV) breaths and pressure support ventilation (PSV).

Clinical and demographic profile at the time of admission to ICU including age, sex, smoking status, history of previous hospital admissions, history of previous intubation and/or ventilatory support, prior evidence of cor pulmonale with or without congestive heart failure were recorded. Findings on clinical examination including heart rate, respiratory rate and mean blood pressure were recorded. Acute physiology and chronic health evaluation II (APACHE II) score and Glasgow coma scale (GCS) were recorded at the time of admission to the ICU. Acid-base abnormalities at the time of presentation were analyzed by recording the arterial blood gas analysis and serum electrolytes (estimations done on AVL 995S). Renal functions, liver functions and serum albumin (SA) done at the time of admission were also recorded. Development of complications during mechanical ventilator such as pneumothorax and ventilator associated pneumonia (VAP) were recorded. Development of acute respiratory distress syndrome (ARDS), sepsis and multi-organ failure was also documented. ARDS was defined as presence of bilateral pulmonary infiltrates on chest radiograph in presence of hypoxemia with PaO_2 _/ FiO_2 _ratio less than 200 without any evidence of left atrial hypertension (American-European Consensus Conference) [5]. Sepsis was defined as the presence of a clinically identified site of infection (*eg*, pneumonia) and two or more of the following: temperature > 38°C or < 36°C; heart rate > 90 beats/min; respiratory rate > 20 breaths/min or PaCO_2 _< 32 mm Hg; and WBC count > 12 × 10^9^/L, < 4.0 × 10^9^/L, or > 0.10 immature forms (*ie*, bands) (American College of Chest Physicians/Society of Critical Care Medicine Consensus Conference) [6]. Days on ventilator, days of ICU stay and days of hospital stay were recorded for all the patients. Primary outcome measure was hospital mortality.

### Statistical analysis

Data were double entered to minimize errors and managed on an 'Excel' master sheet. Analysis was done using the statistical software '*SPSS version 10.0*' (SPPS Corp, Chicago, IL, USA). Descriptive analysis consisted of mean with standard deviation and range for various parameters. Study group was split on the basis of final outcome. Various parameters were compared between the two groups to identify the predictors of mortality. Continuous variables were analyzed using student's t-test whereas Fisher's exact test was used to compare the ordinal variables. Baseline parameters significant on univariate analysis at p < 0.1 were identified as potential predictor variables. These parameters were evaluated using multivariate logistic regression analysis (backward stepwise method) to determine independent predictors of mortality. An equation was constructed using the independent predictors based on the adjusted odds ratios and a diagnostic rule was defined. To evaluate the predictive capability of the variables and the equation, receiver-operator characteristic (ROC) curves were constructed with sensitivity (on X-axis) and 1-specificity (on Y-axis) for various cut-offs. Significance was considered at p < 0.05 (only two tailed) for the present study.

## Results

### Baseline characteristics

Demographic and baseline clinical and laboratory profile of the study group are presented in Table 1. Almost all patients had type II respiratory failure (n = 74, 90.2%) and showed acute on chronic respiratory acidosis. Study cohort mostly consisted of critically ill patients as suggested by a high mean APACHE II score. History of smoking could be elicited in 65 patients (79.3%). A significant number of patients had history of previous hospitalization as well as intubation (39% and 18.3% respectively). Almost 55% of the patients (n = 45) had evidence of underlying cor pulmonale. Fifteen patients (18.3%) had underlying diabetes mellitus whereas 12 patients (14.6%) were on treatment for hypertension. None of the patients suffered from any other co-morbid condition. An attempt was made to define the cause of exacerbation for all patients. There was evidence of pneumonia in 67% (n = 55) of patients whereas pneumothorax was responsible for decompensation in 3 patients (3.7%). No obvious cause could be found in 24 patients (29%). Only one patient had evidence of sepsis, but none had ARDS at the time of admission to the ICU.

**Table 1:** Descriptive profile of the study group (n = 82)

	**Minimum**	**Maximum**	**Mean ± Std. Deviation**
Age (years)	35	85	60 ± 10
APACHE II score	3	33	13 ± 6
PR (per minute)	46	166	105 ± 19
RR (per minute)	10	46	27 ± 10
MBP (mmHg)	20	126	89 ± 19
GCS	3	15	12.1 ± 3
pH	6.87	7.44	7.25 ± 0.19
PaCO_2 _(mmHg)	40.7	130.7	76.6 ± 23.5
PO_2 _(mmHg)	31.5	142.3	83.9 ± 41.7
HCO_3 _(mmHg)	5.4	55.4	32.3 ± 8.7
Serum Na (mEq/L)	115	152	136 ± 7
Serum K (mEq/L)	2.00	6.80	4.2 ± 0.9
Serum Albumin (gm%)	1.7	4.4	3.2 ± 0.7
Days on ventilator	1	33	8.7 ± 4.6
Days of ICU stay	1	35	9.6 ± 6.2
Days of hospital stay	1	63	16.3 ± 10.4

RR: Respiratory rate, PR: Pulse rate, MBP: Mean blood pressure, GCS: Glasgow coma scale, Serum Na: Serum sodium, Serum K: Serum potassium.

### Hospital course

Non Invasive Ventilation (NIV) was used as initial strategy in 17 patients (20.7%). This strategy had a success rate of 59% (n = 10). Sixty-nine patients (84.1%) received invasive ventilation (including seven patients who failed NIV and had to be intubated). Sepsis developed in 11 patients (13.4%) and all these patients eventually died. Parameters associated with development of sepsis were high APACHE II score (18 vs. 12, p = 0.005) and low SA (2.6 gm/dL% vs. 3.3 gm/dL, p < 0.001). VAP developed in 6 patients (8.7%) and was associated with an increased stay in the ICU (18 days vs. 10 days, p = 0.021) as well as increased stay in the hospital (30 days vs. 15 days, p = 0.005). Outcome was not significantly affected by development of VAP (50% versus 42.8%).

### Outcome

**Figure 1:**
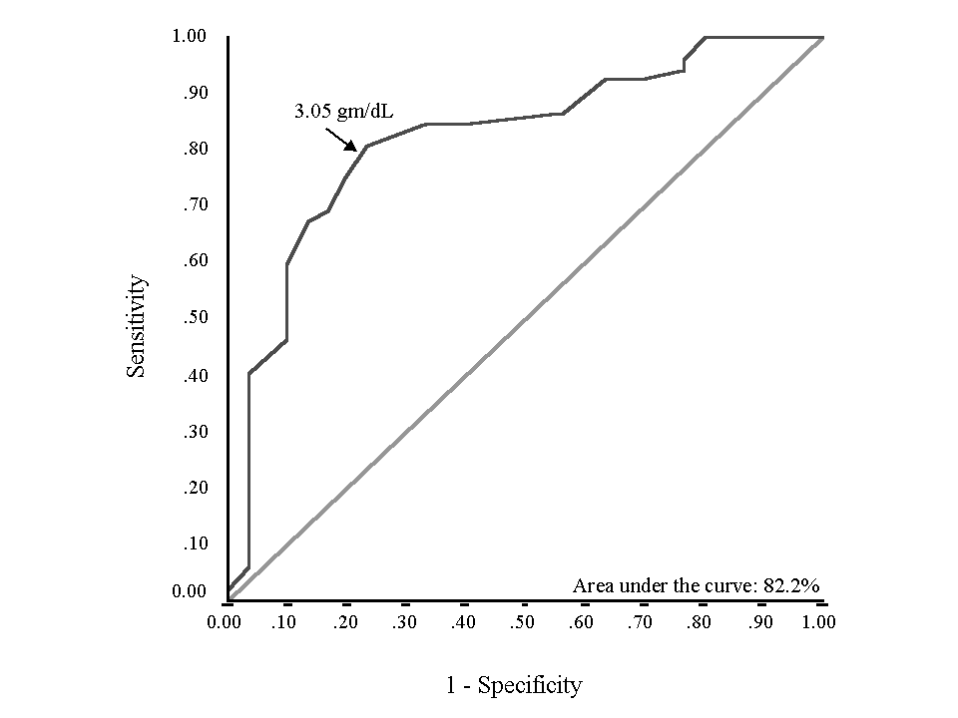
Receiver operator characteristic (ROC) curve plotted for studying the diagnostic utility of Serum Albumin in predicting outcome of patients. The choice of cut-off is shown by an arrow (3.05 g/dL).

**Figure 2:**
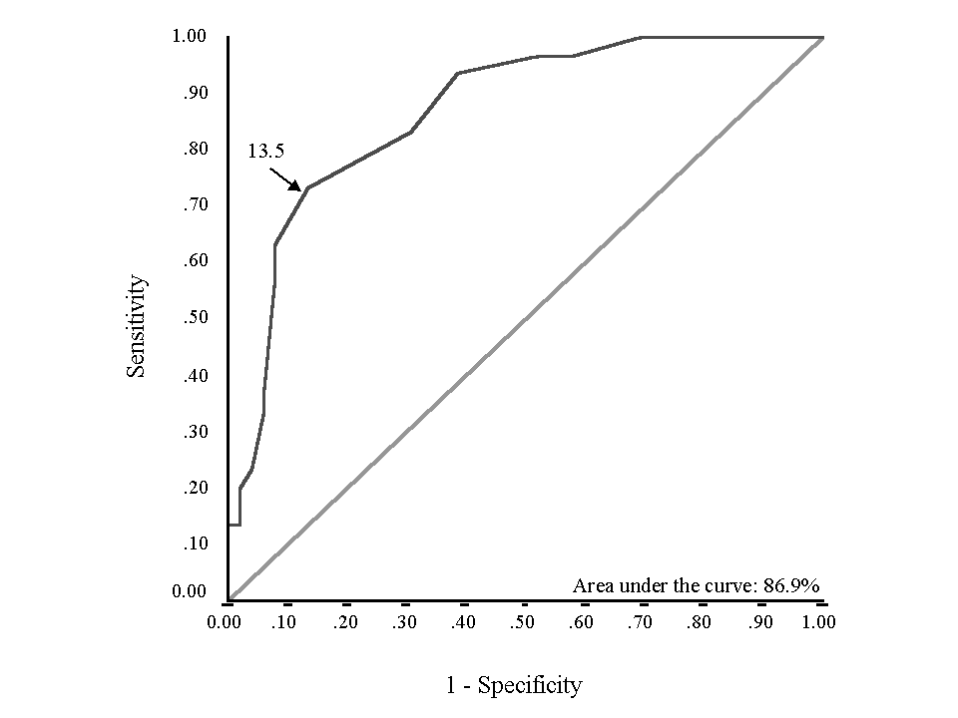
Receiver operator characteristic (ROC) curve plotted for studying the diagnostic utility of APACHE II score in predicting outcome of patients. The choice of cut-off is shown by an arrow (13.5).

**Figure 3:**
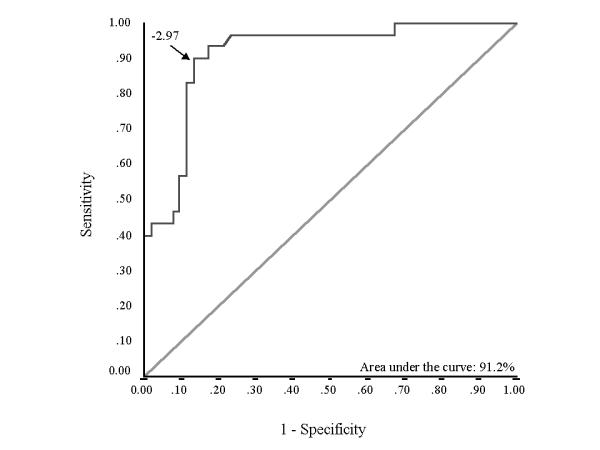
Receiver operator characteristic (ROC) curve plotted for studying the diagnostic utility of score derived form equation in predicting outcome of patients. The choice of cut-off is shown by an arrow (-2.97).

Hospital mortality was 36.6% (n = 30). Various parameters were compared for survivors and non-survivors (table 2). In addition to demographic characteristics (age and sex), presence of cor pulmonale and cause of exacerbation of COPD, baseline parameters significantly different between the two groups on univariate analysis were included in a multivariate equation. APACHE II score at admission to the ICU {odds ratio (95 % CI): 1.32 (1.138-1.532); p < 0.001} and SA (done within 24 hours of admission) {odds ratio (95 % CI): 0.114 (0.03-0.432); p = 0.001} emerged as the independent predictors of mortality. ROC curve showed that both these variables have good predictive capability with area under the ROC curve (AUC) of 86.9% for APACHE II score (Figure 1) and 82.2% for SA (Figure 2). Best cut-off, taken as the value on the ROC curve at the point where curve sharply angulated, was 13.5 for APACHE II score and that for SA was 3.05 gm/dL. Following equation was determined by combining the two variables using the adjusted odd ratio: Score = (0.278 × APACHE II score) - (2.17 × SA), where APACHE II score is the score at the time of admission and SA (gm/dL) is the level with in the first 24 hours. ROC curve for this equation showed an AUC value of 91.2% (Figure 3). We chose a cut-off of -2.97 for the equation. That is, a patient with a score above -2.97 is likely to die whereas the one with below -2.97 likely to survive. This diagnostic rule had a specificity of 86.5% with a sensitivity of 90%. Positive predictive value for this variable was 79.4% whereas negative predictive value was 93.7%. A cut-off of -0.45 was 100% specific for hospital mortality but sensitivity was only 40%. On the other hand a cut-off of -5.5 gave a sensitivity of 100% with specificity of 33%.

**Table 2:** Predictors of mortality for patients with exacerbation of COPD

**Parameter**	**Survivors (n = 52)**	**Non-survivors (n = 30)**	**p value**
	**Mean ± SD**	**Mean ± SD**	
APACHE II score	10.6 ± 4.3	17.5 ± 5.7	0.001
GCS	12.8 ± 2.1	10.8 ± 3.7	0.003
MBP (mmHg)	93 ± 13.6	82.5 ± 24.7	0.015
PR (per minute)	110.7 ± 16.9	102.2 ± 21.1	0.049
Serum Albumin (gm%)	3.5 ± 0.5	2.7 ± 0.6	0.001
PaCO_2 _(mmHg)	81.2 ± 20.8	68.7 ± 25.8	0.018
HCO_3 _(mmol/L)	33.8 ± 8.1	29.6 ± 9.1	0.035
Need of reintubation	35.3%	4.4%	0.001
Renal Failure	Nil	16.7%	0.002
Sepsis	Nil	36.7%	<0.001

GCS: Glasgow coma scale, MBP: Mean blood pressure, PR: Pulse rate.

## Discussion

Primary outcome measure of the present study was hospital mortality. Overall mortality rate was 36.6%. There was a high incidence of need of MV (84.1%). In studies that have taken into account all the patients with COPD requiring hospitalization, mortality rate has been to the tune of 6-42% [7-10]. Weiss & Hudson [11] reviewed 11 studies carried out to study outcome of patients with exacerbation of COPD and found the combined mortality rate to be 20.3%. Selection bias in the inclusion of patients for the present study precludes the generalization of these figures for patients with exacerbation of COPD requiring hospitalization from India. Only a fraction of all the patients with exacerbation of COPD admitted to our hospital are managed in ICU. Many other patients with acute exacerbation of COPD, especially those who do not require ventilatory support, are managed in the wards only. Because of this fact, by including patients who were admitted to ICU the sickest group of patient with exacerbation of COPD was selected.

Various physiological parameters estimated at the time of presentation were analyzed to find predictors of mortality. Only two parameters, namely APACHE II score at admission to ICU and SA in the first 24 hours of admission, were found to be independent predictors of hospital mortality. The same two parameters also predicted development of sepsis on bivariate analysis. Some of the earlier studies have found blood gas parameters like pH [12] and PaCO_2 _[13] to be useful in predicting outcome in COPD patients, whereas others [14-16] did not. In the present study, although PaCO_2 _and HCO_3 _were not independent predictors of mortality they tended to be lower in patients who died and the difference was statistically significant on bivariate analysis. Also, mean pH was similar for the two groups. This has not been reported in the earlier studies and investigators in the past have mostly found high PaCO_2 _levels to be associated with worse outcome. A possible reason for this finding is that patients with hypercapnia with concordantly high HCO_3 _are usually taken care of by mechanical ventilation. On the other hand, low mean PaCO_2 _and HCO_3 _levels in non-survivors probably reflected underlying metabolic acidosis. It has been reported earlier also that, for similar level of acidosis, patients with respiratory failure resulting in respiratory acidosis have better outcome as compared to patients with metabolic acidosis, that is commonly secondary to associated non-pulmonary organ failure [17]. Mean pH was similar for both survivors and non-survivors but survivors comprised predominantly of patients with respiratory acidosis (higher PaCO_2 _as well as HCO_3_) whereas non-survivors consisted of patients with metabolic acidosis (lower PaCO_2 _and HCO_3 _but similar pH). Another finding that corroborates the same fact is that all patients, who had associated renal failure and/or sepsis, died. The incidence of these two complications was significantly higher in non-survivors (renal failure 16.7% vs nil, p = 0.002; sepsis 36.7% vs nil, p < 0.001). Patients with both these complications commonly have associated metabolic acidosis.

Prognostic utility of APACHE II score has been extensively investigated. It has been found useful for prognosticating critically ill patients across a wide array of diagnostic categories. Earlier studies have also found APACHE II score to be useful in predicting mortality in COPD patients with acute exacerbation [18-21] although the timing of scoring after admission has varied in different studies. For example Nevins & Epstein [18] found APACHE II score at 6 hrs after initiation of ventilation to be a useful predictor of mortality. In the present study, APACHE II scoring done at the time of admission to medical ICU was analyzed. SA estimated with in first 24 hrs of admission was also found to be a strong predictor of mortality. SA has also been reported to be of good prognostic value in the past [21-23]. Utility of prognostic value of SA in patients with COPD is interesting. Albumin has a long half-life of approximately 18 days and because of this fact it is unlikely to change with development of acute respiratory failure in patients with COPD. On the other hand SA is known to reflect the underlying nutritional status and to be affected by the severity of chronic illness. These factors are of obvious significance in deciding the outcome of these patients.

An important purpose of the present study was to define predictors, which could help to identify patients that are likely to have worse outcome. This would help us to segregate patients who need to be managed aggressively from the very beginning. We looked at individual predictive utility of the parameters (SA and APACHE II score) that were found to be independent predictors of mortality. Both these parameters had good predictive value as evidenced by high AUC values. To improve the predictive utility, an equation was constructed using the adjusted odds ratio of the two parameters. ROC curve for this equation had a superior AUC value of 0.912. A good prognostic marker needs to be highly specific so that false positives remain low. On the other hand, good sensitivity is also desirable so that false negatives are not too high. A cutoff value of -2.97 has been suggested, which is associated with good specificity (86.5%) as well as sensitivity (90%) for predicting mortality of these patients. Cut-off that were associated with 100% specificity and sensitivity were also determined as different ICU's across the globe may have different priorities at different times.

Although prospective studies are required to validate the findings of present study, an equation devised by combination of APACHE II score and SA appears to make sense. Estimation of APACHE II score makes use of various physiological variables but does not include SA levels. Also, the chronic physiology score in APACHE II fails to stratify patients according to varying severity of chronic illnesses. This tends to happen in patients with COPD as well. Use of SA, which predominantly reflects the severity of chronic illness, in the equation seems to complement the predictive capability of APACHE II score. The results of the present study reflect the complex interplay of factors that occurs in patients with exacerbation of COPD. In these patients, an acute insult in the form of exacerbating illness develops on top of a chronic smoldering illness. Severity of both acute insult as well as the underlying disease in the background of the level of nutritional status tends to determine the outcome of these patients.

Although the equation is useful in to identifying patients with exacerbation of COPD who are likely to have poor outcome, it cannot be looked at in isolation. Other particulars of these patients such as associated illnesses and co-morbidities must be kept in mind before taking a final decision. It cannot be overemphasized that given the sensitivity and specificity of the equation, certain patients with a score below the suggested cut-off may also be sick. Also, the state of patients with exacerbation of COPD tends to remain in a constant flux and need constant monitoring. In spite of having a low score at presentation many of these patients may deteriorate during hospital stay.

It is concluded that APACHE II score at admission and SA levels with in first 24 hrs after admission are independent predictors of mortality for patients with exacerbation of COPD. The equation derived by combining these two parameters is useful for identifying patients that are likely to have poor outcome.

## Competing interests

The author(s) declare that they have no competing interests.

## Authors' contributions

GCK: concept and design of study, management of patients, preparation of the manuscript. AB: concept of the study, management of patients, statistical analysis, preparation of the manuscript. SKS: management of patients and critical review of the manuscript.

## Pre-publication history

The pre-publication history for this paper can be accessed here:


